# Atomic mutagenesis at the ribosomal decoding site

**DOI:** 10.1080/15476286.2016.1256535

**Published:** 2016-11-14

**Authors:** Pius Schrode, Paul Huter, Nina Clementi, Matthias Erlacher

**Affiliations:** Division of Genomics and RNomics, Medical University of Innsbruck, Innsbruck, Austria

**Keywords:** Atomic mutagenesis, mRNA decoding, *in vitro* translation, reconstitution, ribosome

## Abstract

Ribosomal decoding is an essential process in every living cell. During protein synthesis the 30S ribosomal subunit needs to accomplish binding and accurate decoding of mRNAs. From mutational studies and high-resolution crystal structures nucleotides G530, A1492 and A1493 of the 16S rRNA came into focus as important elements for the decoding process. Recent crystallographic data challenged the so far accepted model for the decoding mechanism. To biochemically investigate decoding in greater detail we applied an *in vitro* reconstitution approach to modulate single chemical groups at A1492 and A1493. The modified ribosomes were subsequently tested for their ability to efficiently decode the mRNA. Unexpectedly, the ribosome was rather tolerant toward modifications of single groups either at the base or at the sugar moiety in terms of translation activity. Concerning translation fidelity, the elimination of single chemical groups involved in a hydrogen bonding network between the tRNA, mRNA and rRNA did not change the accuracy of the ribosome. These results indicate that the contribution of those chemical groups and the formed hydrogen bonds are not crucial for ribosomal decoding.

## Introduction

The ribosome is a multifunctional ribonucleoprotein particle that is responsible for fast and accurate protein synthesis. It has to faithfully translate the 4-letter genetic code into amino acid sequences. The basis for this process is the base-complementarity of the transfer RNA (tRNA) anticodon and the messenger RNA (mRNA) codon at the A-site of the ribosome. About 50 different aminoacyl-tRNAs (aa-tRNA) in complex with the elongation factor Tu (EF-Tu) and GTP constantly surround the translation apparatus in the cell.^1^ Out of this pool the correct aa-tRNA has to be selected at every round of translation. It soon became clear that a fast and accurate selection could not solely be based on the simple base-pairing interaction of the codon and anticodon. Single mismatches within this RNA duplexes would not destabilize the interactions to an extend that could explain the accuracy of the translation process (reviewed in^2^). The concept of “kinetic proofreading” was proposed in the 1970s that could explain how enzymes can increase their fidelity.^3,^^4^ Subsequent pre-steady-state kinetics^5-9^ and single molecule experiments^10^^,^^11^ deepened the knowledge of this mechanism and provided a detailed picture of ribosomal decoding. In addition, studies using streptomycin and other error inducing antibiotics targeting the ribosome revealed that the ribosome must be more than just a passive stage for mRNA and tRNA interaction.^12^ Early biochemical investigations located this stage where the codon and anticodon concur in the 30S subunit.^13^ The development of footprinting techniques exhibited that especially nucleotides G530, A1492 and A1493 of the 16S rRNA were protected from chemical modifications upon binding of an aa-tRNA into the A-site.^13-15^ Additional work revealed that these nucleotides are essential for viability and affect the A-site binding.^14,^^16,^^17^

With the turn of the millennium, high-resolution crystal structures became available disclosing the topography of the decoding site.^18,^^19^ For these studies small ribosomal subunits of *Thermus thermophilus* were crystallized in presence of U6 hexanucleotides as mRNAs and anticodon stem loops (ASLs) were bound to the A-site. It was observed that the nucleotides G530, A1492 and A1493 changed their position considerably upon binding of a cognate tRNA to the A-site, underlining the potential importance of these residues. A1492 and A1493, which in the prospecting ribosome are located in the internal loop of helix 44, rotate out of the helix and point into the A-site forming type I and type II A-minor motifs together with the codon-anticodon helix.^2,^^20^ Simultaneously, a rearrangement of the 30S subunit occurs, termed “domain closure,” thereby tightening the acceptor binding site by rotation of the head toward the shoulder (reviewed in^2^).

**Figure 1.**
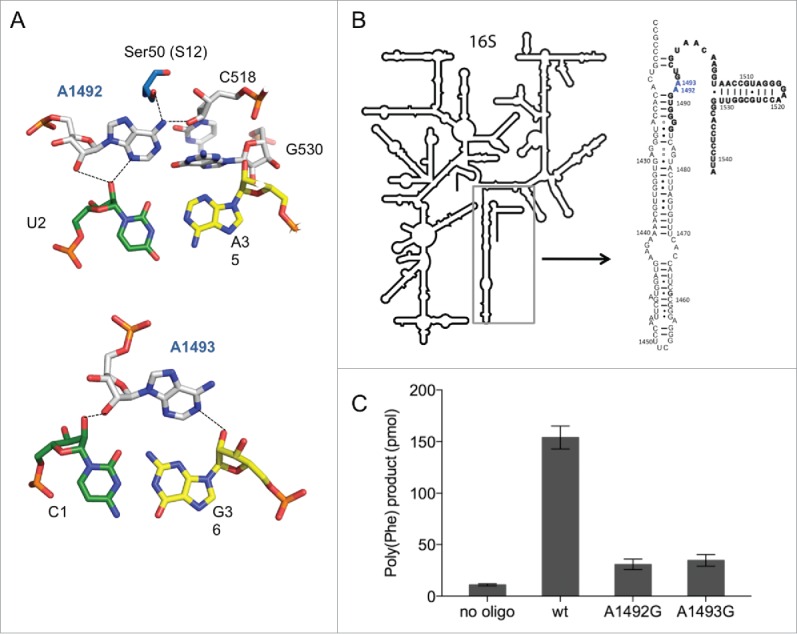
The split 16S rRNA decoding site. (A) Interaction of rRNA with the codon-anticodon helix. 16S rRNA nucleotides are shown in white (A1492, A1493, C518 and G530), mRNA in green (U2) and (C1) and tRNA residues in yellow (A35 and G36). Structures were modified from Demeshkina et al.^19^ (B) The secondary structure of the 16S rRNA used for split 16S rRNA reconstitutions. The oligonucleotide added *in trans* to the assembly reaction is depicted in bold. Nucleotides A1492 and A1493, which were modified in this study are shown in blue. (C) The poly(U) translation activity of ribosomes composed of 12 pmol reconstituted 30S subunits and 5 pmol native *E. coli* 50S. Poly(Phe) product yields of *in vitro* assembled ribosomes in the absence (no oligo) or in the presence of the compensating wild type 57-mer (wt) and carrying A to G mutations at position 1492 or 1493 are depicted. Values are depicted as mean ± SEM from at least 4 independent experiments.

This structural information provided the premises to understand how the ribosome can possibly discriminate between cognate and near- or non-cognate tRNAs. In case a cognate tRNA binds into the decoding site, A-minor motifs are formed. Thereby A1493 spans the minor groove of the tRNA/mRNA helix and contacts both by hydrogen bonding (Fig. 1A). In addition, A1492 interacts with a part of the groove and forms hydrogen bonds with mRNA nucleotides (Fig. 1A). Binding of a near-cognate tRNA, meaning a G-U wobble base pair present either at the first or second position of the codon-anticodon helix, results in a distorted geometry and leads to disrupted hydrogen bonds^2^ This uncompensated loss of desolvation of these hydrogen bonds was postulated not to induce the domain closure of the 30S subunit and therefore the tRNA is rejected.

However, recent X-ray studies using *Thermus thermophilus* 70S ribosomes, harbouring full-length tRNAs and bona fide mRNAs paint a different picture.^21-23^ Mismatches between the codon and the anticodon were positioned at the first or second position of the codon resulting in a G-U base pair. Unexpectedly, a Watson-Crick geometry and not a wobble base pair was formed. This indicated that the hydrogen bonds between G530, A1492 and A1493 and the codon-anticodon helix are not disrupted and therefore do not contribute to the decoding process as proposed in earlier work. Rather recent biochemical and computational investigations confirm these structural observations,^24,^^25^ whereas others assign A1492 and A1493 an active role during tRNA decoding.^26^

In our study we followed the question on the importance of single chemical groups in the decoding site for translational activity and fidelity. An *in vitro* reconstitution approach based on the procedure first described in 1973 was chosen to shed light on the exact decoding mechanism.^27^ This setup was further modified to allow the site-specific introduction of non-natural modifications at the nucleotides A1492 and A1493 within the decoding site of the small ribosomal subunit. The modified ribosomes were tested in *in vitro* translation assays for their activity in protein synthesis. Surprisingly, the decoding site turned out to be more flexible than anticipated and tolerated various modifications at nucleosides A1492 and A1493.

In respect to translation fidelity, potentially important hydrogen bonds could be removed without displaying a major impact on the error rate. These results implicate that the hydrogen bonding interactions are not the major discriminating factor to differentiate between cognate and near-cognate tRNAs. This is in line with recent decoding models based on structural investigations.

## Results

### Split 16S rRNA for 30S assembly

To introduce non-natural modifications site specifically, we established a 30S *in vitro* reconstitution system employing split 16S rRNA molecules. A nick was positioned within helix 44 3′ to nucleotide (nt) U1485 resulting in a 2-pieced (split) rRNA molecule: a 1485 nucleotide long 5′ part and a 57 nt short 3′ part (Fig. 1B). The 3′ oligonucleotide started with the nucleotide G1486 and included the nucleotides A1492 and A1493. This short RNA can be chemically synthesized and allows the substitution of single chemical groups at various positions within this oligonucleotide.^28-32^ The synthesized RNA was added to the assembly reaction *in trans* simultaneously with the 5′ part. After the complete reconstitution of the 30S particles, native *E. coli* 50S subunits were added and the activity of the reassociated 70S was tested by using a poly(U)-dependent poly(Phe) translation assay. Depending on the quality of the reconstitution components, we could incorporate up to 30 phenylalanines per ribosome using the unmodified wild-type (wt) sequence at the endpoint of the reaction (Fig. 1C). We observed that the activity of the small subunit was strictly dependent on the presence of the oligonucleotide, indicating that the 3′ end can be added *in trans* (Fig. 1B) and is successfully incorporated into functional small ribosomal subunits. Aminoglycoside antibiotics that bind close to the decoding center, such as paromomycin, neomycin or streptomycin affected the translation process, indicating a correctly assembled decoding site (see below).

In addition, using transcribed split 16S rRNA carrying A to G substitutions at positions 1492 and 1493, resulted in an approximately 10-fold reduction in poly(Phe) synthesis (Fig. 1C and Fig. S1A). These mutations were reported earlier to have a dominant lethal phenotype and to be defective in protein biosynthesis *in vivo* and *in vitro.*^16,^^17,^^33^ The decline in translation activity was in the same order of magnitude using full-length 16S rRNA transcripts harbouring these mutations (Fig. S1B). This indicates that our experimental approach of using a split 16S rRNA recapitulates, on a qualitative level, the previously observed functional performance of genuine 30S subunits during the decoding process.

Additionally, we took into consideration that the absence of natural modifications within the rRNA was reported to have an impact on activity, assembly and also translation fidelity.^34,^^35^ To ensure that the outcomes of our experiments were only caused by the introduced modifications, we always referred our results to reconstituted particles carrying the unmodified wt sequence. The background level, on the other hand, was set by ribosomes reconstituted in the absence of the short RNA fragment, implicating possible contaminations of protein extracts or 50S subunit preparations. To be certain that oligonucleotides carrying modifications that inactivate the ribosome are assembled correctly, we performed filter binding assays measuring the amount of oligonucleotides that are incorporated in the reconstituted 30S subunit. All tested RNA fragments showed similar incorporation into the 30S, implicating that the modifications do not interfere with the overall assembly (Fig. S2).

Furthermore, we reconstituted ribosomes using the unmodified wt RNA oligonucleotide and added equal amounts of RNA carrying an inactivating modification to the same reaction. We expected the poly(Phe) activity to be reduced compared with wt if equal binding is given, indicating that about the half of the 30S subunits were harbouring the inactive RNA modification. This indeed was the case excluding substantial binding differences of the tested modified RNA fragments (data not shown).

## Effects of modified bases on translation

### Position 1492

**Figure 2.**
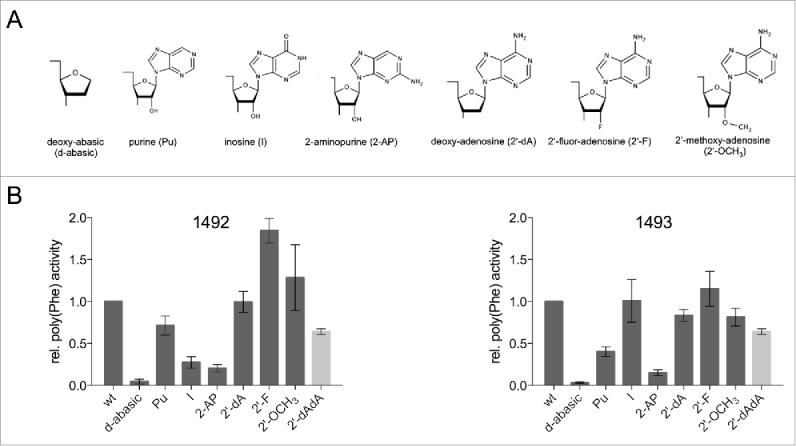
(A) Chemical structures of the tested nucleoside analogs. (B) Product yield of ribosomes carrying modifications at A1492 or A1493 determined in a poly(U) dependent poly(Phe) assay. The activity of ribosomes carrying the unmodified wt RNA oligonucleotide was taken as 1.0. The values shown are the mean ± SEM of at least 3 independent experiments. The bar depicted in gray represents relative poly(Phe) activity for the simultaneous incorporation of 2′-dA at 1492 and 1493.

A1492 forms hydrogen bonds with the mRNA, nucleotides G530 and C518 of the 16S rRNA and serine 50 of ribosomal proteins S12 (Fig. 1A).^19^ We investigated the impact of disrupting single or multiple hydrogen bonds on translation by introducing modified RNA nucleotides (Fig. 2A). In the beginning we concentrated on overall translation activity and took the poly(U) directed poly(Phe) synthesis as indication thereof (Fig. 2B). The most severe chemical and steric modification which was introduced in the decoding site was a deoxy-abasic variant (d-abasic) (Fig. 2A), which eliminated not only all hydrogen bonds but also potential stacking interactions. This change of the decoding site composition drastically reduced the activity to background levels and hardly allowed a reliable quantification of the product formed (Fig. 2B). Single deletions of proposed interaction partners did not reveal equally strong effects. The incorporation of purine (Pu), lacking the amino group at position 6, led to a slightly reduced activity, although the amino group is in hydrogen bonding distance to C518 and Ser(50) of S12. However, positioning a carbonyl oxygen at position 6 (incorporating inosine (I)) showed an approximately 5-fold reduction (Fig. 2B). The introduction of 2-aminopurine (2-AP) at 1492 significantly reduced the amount of product formed as well. Both of these modifications seem to explain why G mutants are not able to efficiently translate the poly(U) message. Neither inosine nor the 2-aminopurine was tolerated in the decoding site whereas the lack of an exocyclic group at position 6 of adenine did not drastically affect protein biosynthesis.

Also the 2′-OH group of the ribose at position 1492 was modified. This group is in hydrogen bonding distance with the second nucleotide of the mRNA codon in the A-site. An incorporation of a deoxy-adenosine (2′-dA) retained almost full activity. Even the introduction of a bulky methoxy-group (2′-OCH_3_) reduced the amount of poly(Phe) only slightly, pointing to a certain flexibility concerning the 2′ position of A1492. Positioning a fluor-atom at the 2′ position (2′-F) even increased the amount of peptide formed (Fig. 2B).

### Position 1493

A1493 spans the minor groove of the codon-anticodon helix and interacts with both the mRNA and the tRNA by hydrogen bonds (Fig. 1A). Again we introduced nucleotide derivatives at position 1493 to alter the composition of the decoding site. In line with the results at position 1492 the deoxy-abasic derivative in the A-site almost completely eliminated ribosomal activity (Fig. 2B). However, introducing a purine at 1492 reduced the product yield more than 2-fold, which is unexpected considering that no obvious hydrogen bonding partners were evident from the crystal structures (Fig. 2B). Incorporating an inosine did not reduce the amount of formed peptide significantly, rescuing the activity resulting from the absence of the N6 amino group. Additionally, the introduced 2-aminopurine hampered the ribosome to translate the poly(U) message. This modification reduced the activity by a factor of 10, reminiscent of the A to G substitution at this position. We also examined the effect of dA-incorporation at 1493 and determined only a small loss of product formation. Positioning hydrogen bond acceptors like fluorine at the 2′ position resulted in a fully active translation apparatus. The rather bulky methoxy group only modestly reduced the translation activity.

Because the 2′-OH of A1492 and A1493 were both supposed to be involved in multiple hydrogen bonds and single deletion of these hydroxyl groups did not show strong defects in poly(Phe) synthesis, deoxy-adenosines at position 1492 and 1493 (2′-dAdA) were simultaneously introduced. Even these modified ribosomes showed considerable amounts of product formed (Fig. 2B).

Additionally to using a poly(U) message, an mRNA carrying a Shine Dalgarno (SD)-sequence as well as multiple UUC codons were employed. In analogy to the poly(U) mRNA this SD-(UUC)_12_ -mRNA did not carry a start codon but an efficient translation of this mRNA requires a faithful reading frame maintenance because of the absence of the amino acids corresponding to the frameshifted context. The amount of product formed was significantly less (about 30 fold) than using a poly(U) message as was also described for native 70S particles.^36^ However, these results in principal reflected those obtained by using the standard poly(U) dependent translation assay suggesting an unperturbed reading frame maintenance using assembled 30S harboring split 16S rRNA (Fig. S3).

### Rescue of abasic variants using paromomycin or streptomycin

**Figure 3.**
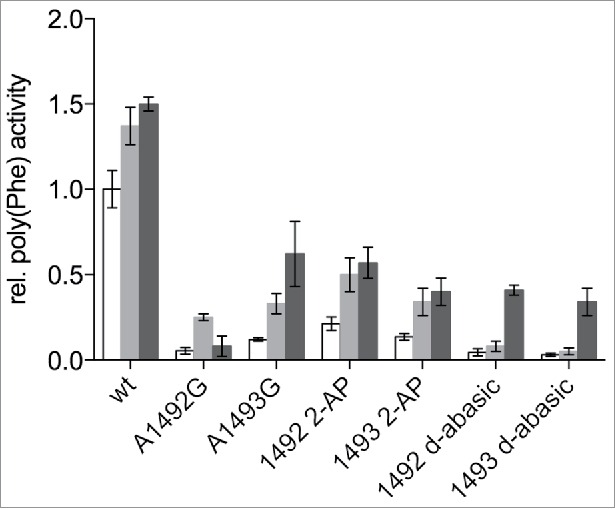
Effects of streptomycin and paromomycin on modified reconstituted ribosomes. Paromomycin (gray) and streptomycin (black) were added to a final concentration of 5 µM. The poly(Phe) translation activity of wt ribosomes without antibiotics (white) was set to 1. The values are means ± SEM of at least 3 independent experiments.

Aminoglycoside antibiotics are known to be able to compensate for the loss of interactions in the A-site when using mRNA codons harbouring 2′-deoxynucleotides.^25^ In addition, ribosomes carrying either A1492G or A1493G mutations can be partly recovered from their defects in tRNA binding into the A-site, peptide bond formation or EF-Tu dependent GTP hydrolysis.^25,^^33^ We tested if the aminoglycoside antibiotics paromomycin or streptomycin could rescue the hampered ribosomes carrying the deoxy-abasic variant or the 2-AP in the decoding site. The addition of paromomycin to reconstituted ribosomes carrying the unmodified wt sequence, stimulated poly(Phe) synthesis (Fig. 3). This was observed earlier using a poly(U) based translation system using native ribosomes as well.^36^ The abasic variants, showing no product formation in the absence of aminoglycoside antibiotics, could be partially activated by paromomycin, as were 2-AP harbouring ribosomes. Essentially the same effects but to a smaller extend could be seen by adding streptomycin, which has a binding site distinct to paromomycin and a different mechanism of interacting with the ribosome.^37,^^38^

### Translation fidelity of modified ribosomes

**Figure 4.**
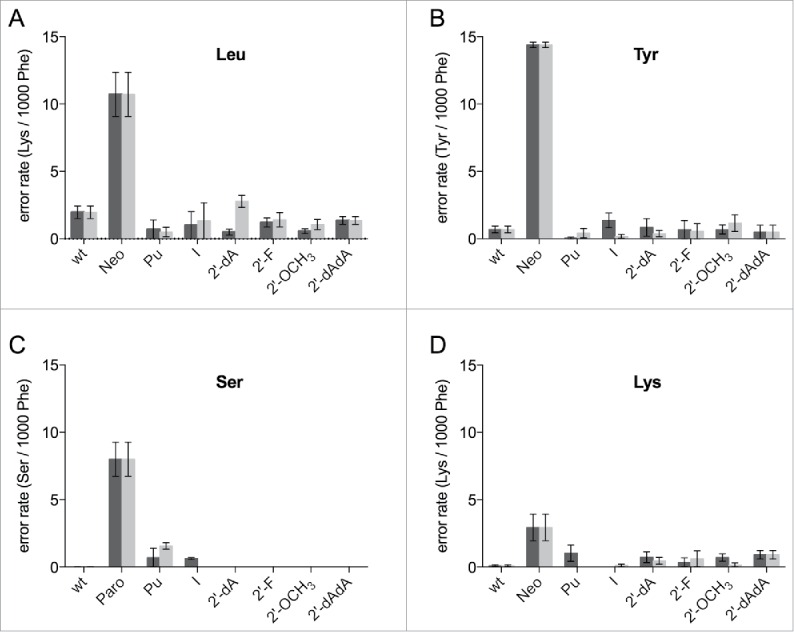
Misincorporations of various amino acids in a poly(U) based translation system. Ribosomes were reconstituted harboring modifications at position 1492 (dark gray) and 1493 (light gray) of the 16S rRNA. The error rates per ribosome per 1000 phenylalanines (Phe) translated were determined for leucine (A), tyrosine (B), serine (C), and lysine (D), respectively. The antibiotics neomycin (Neo) or paromomycin (Paro) served as positive controls. The mean ± SEM of at least 3 independent experiments are shown.

By chemically modifying the decoding site we wanted to additionally elucidate the effect on translation fidelity. We assumed that eliminations of potential hydrogen bonding partners or sterical modifications might significantly interfere with the integrity of the decoding center and consequently reduce the capability to accurately discriminate tRNAs. Classically, leucine is the amino acid most frequently misincorporated when translating a poly(U) mRNA. The Leu codon differs from the Phe codon only in the third position, which is not as tightly monitored by the ribosome as the first 2 positions. The error rate of reconstituted ribosomes carrying the unmodified sequence was ∼1.8 misincorporations/1000 phenylalanines (Fig. 4A). Compared to literature we detected higher numbers of misincorporations, which is likely to be caused by higher concentrations of Mg^2+^, that are needed to achieve efficient translation activities using *in vitro* assembled ribosomes.^36^ The addition of neomycin or paromomycin, members of the aminoglycoside family of antibiotics, increased the number of misincorporations and validated this modified experimental setup (Fig. 4A-C).

All modifications at 1492/93 that showed considerable translation activities were examined employing this misincorporation assay. 2-AP and the d-abasic could not be investigated due to their low translation activity. Strikingly, neither modifications at the ribose nor at the base significantly reduced the fidelity of the ribosomes (Fig. 4A). Because A1492 and A1493 are not supposed to be directly involved in monitoring the wobble position of the codon, we also wanted to determine if other amino acids are effected by the modified decoding site. The amount of L-serine, L-tyrosine and L-lysine being misincorporated into a poly(Phe) peptide using a poly(U) message, was determined. It was reported that mutations of the 16S rRNA, which induce misreading are codon dependent,^39^ hence also modifications at the decoding nucleotides A1492 and A1493 might have codon-anticodon dependent effects.

We quantified the wrongly incorporated tyrosine, which is encoded by UAC and UAU, placing a U-U mismatch at the second position. For the unmodified reconstituted particles an average error rate of ∼0.6 Tyr/1000Phe was measured (Fig. 4B). None of the introduced modifications did increase the error rate considerably. To further examine the decoding performance we also utilized L-serine having a U-G mismatch at the second codon position to be analyzed. In line with our results employing leucine and tyrosine, strong defects in tRNA discrimination were not observed (Fig. 4C). Interestingly, ribosomes carrying a purine at 1492 or 1493 or harbouring inosine at 1492 showed modestly higher misincorporations of serine into the poly(Phe) peptide chain.

Finally, we determined the misincorporation of lysine, which can only be incorporated overcoming 3 non Watson-Crick interactions. The tested modifications did not distinctively alter the performance of the ribosome. However, some modifications at 1492 did show a slightly higher number of wrongly incorporated lysines (Fig. 4D). Because of the low error rate in general (approximately 0.1 Lys/1000 Phe) these effects have to be considered cautiously. Even in the presence of aminoglycoside antibiotics the misincorporation of lysine was low compare with the other amino acids tested (Fig. 4).

### Discussion

In this study we established a novel *in vitro* 30S reconstitution system to modulate the chemical and sterical composition of the ribosomal decoding site. By employing a chemical synthesized RNA oligonucleotide complementing a shortened *in vitro* transcribed 5′ fragment for reconstitution of the 30S subunit, it is possible to site-specifically introduce different types of nucleoside modifications at position 1492 and 1493 (Fig. 1B). This allowed investigating the influence of single chemical groups of the decoding site on translation activity and translation fidelity.

A1492 and A1493 were shown to be essential for ribosomal function^40^ and mutations and deletions of these nucleotides severely hampered translation.^14,^^16,^^17^ Deoxy-abasic sites at position 1492 and 1493 were introduced, to eliminate most of the potential interaction partners possibly involved in hydrogen bonding or base-stacking interactions. In contrast to a complete deletion of a nucleotide, the backbone is still intact and the spacing between the neighboring nucleotides should not be altered. Additionally, the ribose ring itself could also be contributing to the functionality.^28^ However, ribosomes encompassing abasic sites did not show translation activity (Fig. 2A), underlining the necessity of the base during peptide synthesis. Aminoglycoside antibiotics like paromomycin could partially recover the inactive ribosomes (Fig. 3) in a manner observed with mutations at 1492 and 1493^33,^^38,^^41^ indicating that the presence of the bases is not absolutely essential to induce domain closure, if paromomycin is bound. This agrees with recent crystal structures that paromomycin binds the phosphate backbone of 1493 and thereby modulates ribosomal functions.^22^

To dissect the decoding process more precisely, we introduced adenine derivatives differing only at single positions from the unmodified RNA (Fig. 2B). Interestingly, amino groups at the C2-position at 1492 and 1493 seem to sterically interfere with the geometry of the decoding site by disturbing the A-minor motif.^20,^^33^ Stacking effects are improbable to be responsible because of a similar π-electron distribution throughout the aromatic ring system within adenosine and 2-aminopurine.^29^ Unexpectedly, the amino group at A1492 could be eliminated without drastic effects on peptide synthesis. According to the crystal structure this group is in hydrogen bonding distance to Ser50 and C518. It is possible that the interaction of N3 with U(+5) is sufficient to provide the correct geometry. However, an exocyclic oxygen at C6 either disturbs the correct formation of a functional decoding site or interferes with translocation as this group is involved in the interaction with EF-G.^42^

According to structural data, the amino group at 1493 was not found to be interacting with neighboring residues of the decoding site (Fig. 1A). Nevertheless, the loss of this group strongly reduced translation, but the activity could be rescued by placing a carbonyl oxygen at this position (Fig. 2). This indicates that a hydrogen bond acceptor is needed to form a productive interaction with EF-G and consequently for translocation.^42^

The formation of hydrogen bonds between the 2′-OH groups of A1492 and A1493 and the mRNA was proposed to be an important part of the decoding process.^2,^^19^ Employing deoxy-adenosines in the decoding center allowed us to determine the importance of these interactions during translation. Strikingly, the effects observed were modest (Fig. 2). Even a simultaneous deletion of the 2′-hydroxyl groups at 1492 and 1493 did not fully inhibit the translation activity of the ribosome. This is in agreement with recent studies where 2′-deoxy or 2′-fluorine nucleotides were introduced into mRNAs thereby eliminating its interactions with A1492 and A1493.^25^

Being able to modulate the decoding site, we also investigated the decoding fidelity of the modified ribosomes. Leucine is the classic amino acid to be misincorporated at UUU, since the Leu codons (UUA and UUG) only differ from the Phe codon at the wobble position. Independent of the modification we introduced at either 1492 or 1493, an increase in leucine incorporation could not be observed. Even the simultaneous introduction of dA1492 or dA1493 did not reduce the translation accuracy. These results indicate that the hydrogen bonds formed by adenosine at 1492 or 1493 with the mRNA or tRNA in the decoding site are not essential for faithful translation.

Because it was shown that mutations of the 16S rRNA that influence translation fidelity to varying degrees depend on the codon-anticodon interaction,^39^ different amino acids were tested for their ability to be wrongly embodied in a poly(Phe) peptide. For the incorporation of tyrosine a U-U mismatch at the second codon position is present but none of the modifications tested made the ribosome more error-prone (Fig. 4B). The same held true for a serine incorporation placing a U-G mismatch at the second codon position (Fig. 4C). Only purines at 1492 and 1493 or inosine at 1492 seem to increase the error rate slightly, indicating a potential involvement of the exocyclic N6 during decoding of this codon-anticodon combination.

When we tested for lysine, which is encoded by AAA and AAG and 3 mismatches have to be overcome, we could determine a slightly increased error rate when incorporating modifications at position 1492. Although the overall error rate that was determined was very low, modifications at the C6 and 2′-ribose position seem to slightly lower the decoding fidelity. Possibly, the interactions formed by 1492 are adjuvant to help discriminating certain types of codon-anticodon interactions.

Recent structural data proposed that the geometry of cognate and near-cognate interactions at the decoding site show the same geometry, excluding the possibility to discriminate through differences in the number of hydrogen bonds formed.^21-23,^^43^ These findings based on high-resolution crystal structures were supported by biochemical studies introducing modified RNA nucleotides into the mRNA.^25^ Molecular dynamic studies proposed that the main task of G530, A1492 and A1493 is to exclude water molecules from the codon-anticodon helix, thereby enhancing the discriminating power of the ribosome.^24^ However, at the same time all atom molecular simulation studies argue in favor of hydrogen bonding between A1492/A1493 and the mRNA/tRNA helix being an essential part of the decoding process.^26^

The use of reconstituted 30S subunits to substitute or delete single chemical groups allowed us to evaluate the importance of single interactions of A1492 and A1493 during peptide synthesis. The advantage to manipulate the decoding site at the molecular level comes with the disadvantage of lower translation activities, limiting certain applications. Nevertheless, this approach enabled us to modify 16S rRNA nucleotides in the decoding site and to describe their impact on translation activity and fidelity. We observed that single hydrogen bonds could be removed without having a major impact on translation fidelity, arguing against their general importance during the aa-tRNA discrimination process. However, small defects depending on the codon-anticodon interactions could be detected. This suggests that hydrogen bonding interactions might not be essential but beneficial for discriminating certain codon-anticodon combinations. It seems feasible that the decoding site needs various tools to be able to read every possible codon-anticodon interaction and decide which one to accept and which one to reject.

## Material and methods

### Material

tRNA^Phe^, tRNA^Lys^ and tRNA^bulk^ as well as the poly(U)-mRNA were ordered from Sigma Aldrich. The RNA oligonucleotides and mRNAs used in this study were purchased from Integrated DNA Technology (IDT), Microsynth or Dharmacon. Antibiotics and reagents were dissolved in water, unless otherwise stated.

### Generating 16S rRNA constructs

To create the different 16S rRNA constructs used for reconstitution, transcription templates were generated by PCR and cloned into pUC19 vectors. The full-length 16S rRNA gene was amplified from genomic DNA of *E. coli* CAN20–12E using a forward primer GG**CTGCAG**GATCCTAATACGACTCACTATAGGGAAATTGAAGAGTTTGATCATGGCTCAGATTG including a T7 promoter sequence (underlined) and the PstI restriction site (bold). Two additional Gs following the T7 promoter were introduced during PCR to ensure efficient transcription initiation. The reverse primer for this PCR with the sequence CCC**GGATCC**TAAGGAGGTGATCCAACCGCAGGTTC contained a BamHI site (bold) to enable run-off transcriptions. The PCR product was gel-purified and subsequently cloned into a pUC19 vector using the indicated restriction sites. The 16S rRNA-1485 was generated similarly to the full-length 16S rRNA construct but using a different reverse primer CCCC**TCTAG**AGTCATGAATCACAAAGTGGTAAGC containing an XbaI restriction site. This transcription template allowed the generation of the 16S rRNA terminating at position 1485. Transcriptions were performed using the RiboMAX™ Large Scale RNA Production System-T7 (Promega) according to the manual. The transcript was purified as previously described.^30,^^44^ The RNA oligonucleotides compensating the missing 3′ end of the 16S rRNA with the sequence GGGGUG**AA**GUCGUAACAAGGUAACCGUAGGGGAACCUGCGGUUGGAUCACCUCCUUA were chemically synthesized. The nucleotides corresponding to position 1492 and 1493 are depicted in bold.

### 30S *in vitro* reconstitution

The 30S subunits were assembled using 12 pmol 16S rRNA-1485, 100 pmol of the RNA oligonucleotide and total proteins of the 30S subunits in 1 x assembly buffer (25 mM Tris/Cl pH 7.5, 330 mM KCl, 2 mM DTT).^45^ For reconstitutions using full-length 16S rRNA the MgCl_2_ concentration was set to 20 mM and for the split 16S rRNA to 30 mM. The total proteins (TP30) were prepared according to a modified protocol from.^46^ The last dialysis step of the protein preparation was performed against the buffer TKMD (25 mM Tris/Cl pH7.5, 1M KCl, 20 mM MgCl_2_, 2 mM DTT).^45^ The optimal amount of TP30 added to one reconstitution was optimized after every protein preparation. The rRNA and the ribosomal proteins were incubated separately for 20 minutes at 40°C before combining them. After a subsequent incubation step at 40°C for 40 minutes, 5 pmol of purified *E. coli* 50S subunits were added and incubated for 20 minutes. The ribosomal particles were then precipitated employing 3 volumes ethanol and the samples were incubated at −80°C for 45 minutes. After a 30-minute centrifugation step the pellets were resuspended in the reaction buffer needed for subsequent functional testing.

### Poly(U)–dependent poly(Phe) synthesis

The assay was modified from previously used protocols.^36,^^44^ One reaction contained the assembled 70S from one reconstitution (see above). During the first step the ribosomes were dissolved in 15 µl buffer M (20mM Hepes/KOH 7.6, 10 mM MgAc_2_, 150 mM NH_4_Ac, 4 mM 2-mercaptoethanol, 2 mM spermidine, 0.05 mM spermine) containing 20 µg of poly(U). The poly(U)-message was bound to the ribosome for 15 minutes at 42°C. Meanwhile the charging reaction was prepared containing 3.2 mM ATP, 1.6 mM GTP, 1.6 mM acetylphosphate, 1 nmol unlabelled L-phenylalanine, which was combined with [^3^H]-L-phenylalanine (specific activity ∼300 cpm/pmol) and 4-5 µl of *E. coli* S100.^47^ The binding and the charging reactions were combined and incubated for 3 hours at 42°C. Then 30 µl of BSA (10 µg/µl) were added and a hot trichloroacetic acid (TCA) precipitation was performed. Therefore 2 ml of 5% TCA were added and incubated for 15 minutes at 95°C. After cooling the reaction on ice the samples were filtrated through glass microfiber filters (Whatman) and subsequently washed with 2 ml of 5% TCA. The filters were dried using 2 ml of ethanol/ether (50/50) and quantified using a scintillation counter. For the rescue experiments the antibiotics were added after the binding and charging reactions were combined to a final concentration of 5 µM.

### Misincorporation assays

This assay was performed as described for poly(U)-dependent poly(Phe) synthesis with following adjustments. For one reaction 24 pmol of reconstituted 30S particles were associated with 10 pmol of native *E. coli* 50S subunits and treated as described above. Instead of purified tRNA^Phe^ tRNA^bulk^ was added and [^14^C]-L-Phe was employed instead of [^3^H]-L-Phe. L-Leucine (L-Lysine, L-Serine, L-Tyrosine) was ^3^H-labeled and the specific activity was between 7000-15000 cpm/pmol. The reactions were incubated for 3 hours at 42°C. The scintillation counter was programmed to allow the separation of ^3^H and ^14^C signals.

### Translation of SD-(UUC)_12_ mRNA

This assay was performed as described above with the poly(U) mRNA but SD-(UUC)_12_-mRNA with the sequence GCGGCAAGGAGGUAAAUAUUCUUCUUCUUCUUCUUCUUCUUCUUCUUCUUCUUC was used instead.^48^ The specific activity of [^3^H]-L-Phe was increased to 1500 cpm/pmol.

### Quantification of the 57-mer in reconstituted particles

The reconstitution of the small ribosomal subunit was performed in presence of ^32^P-labeled oligonucleotides. After the assembly of the 30S particle, filter binding was performed using nitrocellulose filters (Millipore, MF 0.45 µM HA). The reaction was applied on the filter in 100 µl assembly buffer and washed with 2 ml of the same buffer. The ^32^P-labeled oligonucleotides were quantified using a scintillation counter.

## Supplementary Material

Supplemental_Figures_1-3.docx
