# Supplementary material for: Atomic mutagenesis at the ribosomal decoding site
Source: RNA Biol. 2016 Nov 14;14(1):104–12. doi: 10.1080/15476286.2016.1256535 (PMC5270523; doi:10.1080/15476286.2016.1256535)
Supplement: Supplemental_Figures_1-3.docx [file krnb-14-01-1256535-s001.docx]

**Supporting Information**

**Atomic mutagenesis of the ribosomal decoding site**

Pius Schrode^1#^, Paul Huter^1#^, Nina Clementi^1^ and Matthias Erlacher^1,^*

^1^ Division of Genomics and RNomics, Medical University of Innsbruck, 6020 Innsbruck, Austria,

* To whom correspondence should be addressed. Tel: +43512900370256; Fax: +43512900370100 Email: Matthias.Erlacher@i-med.ac.at

^#^ Contributed equally to this work

**Present Address:** Paul Huter, Department of Biochemistry, Gene Center Munich, University of Munich, 81377 Munich, Germany

**Present Address:** Pius Schrode, Biologics Technical Development and Manufacturing, Sandoz GmbH, 6250 Kundl, Austria


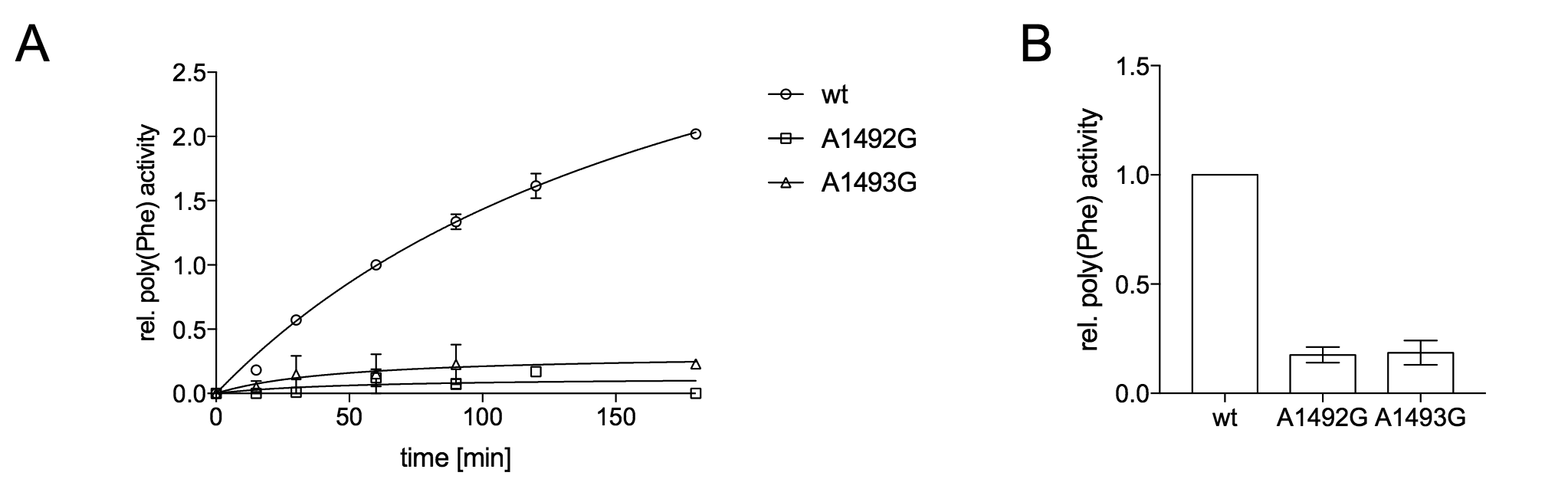


**Supplemental Figure 1**: (A) Timecourse experiment of reconstituted ribosomes harbouring the unmodified wild type and A1492G and A1493G RNA oligonucleotides. The ribosomes were tested in poly(U) dependent poly(Phe) synthesis. The activity derived from particles reconstituted in absence of the complementing RNA oligonucleotide was subtracted. The 60 minutes time point was taken as 1. (B) Translation activity of reconstituted ribosomes harboring full-length 16S rRNA carrying A to G mutations at 1492 and 1493. The values shown are the mean ± SEM of at least 3 independent experiments.


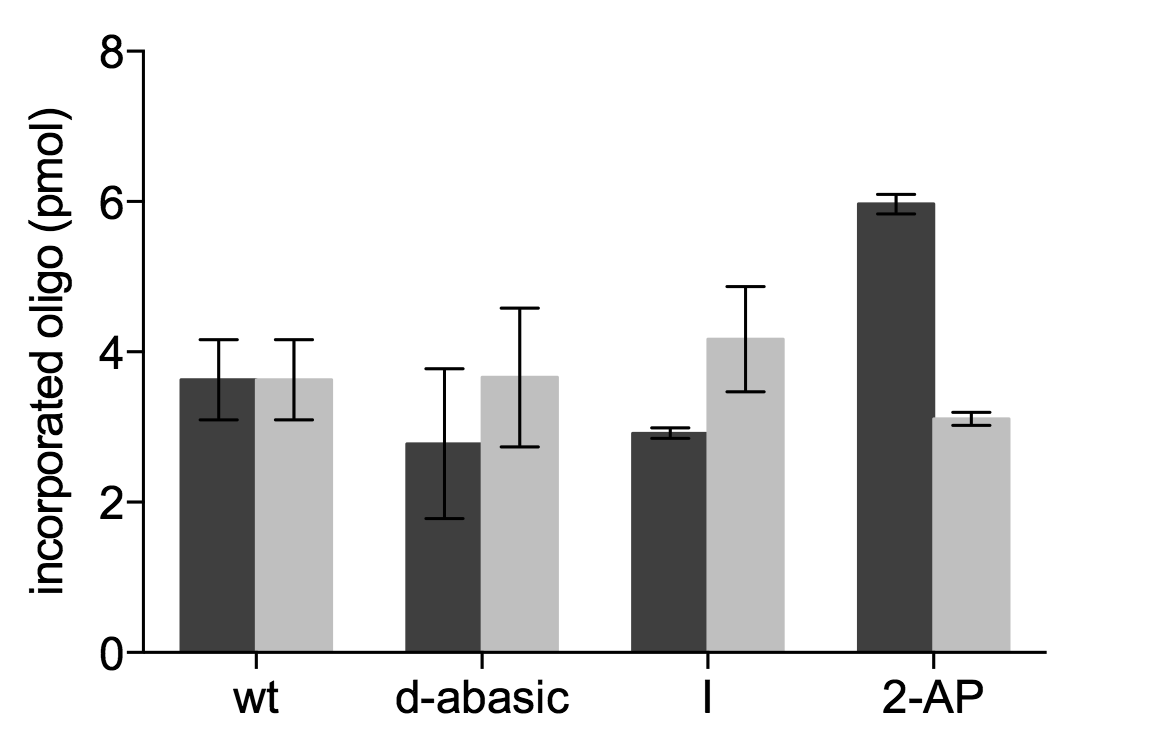


**Supplemental Figure 2:** Quantification of incorporated 57mer during *in vitro* assembly of the 30S subunit. Ribosomes were reconstituted in the presence of ^32^P-labeled RNA oligonucleotides carrying either the unmodified wt sequence or modifications at position 1492 (dark grey) and 1493 (light grey), respectively. For the quantification filter binding was carried out and the amount of RNA oligonucleotides incorporated was determined by liquid scintillation counting. The values shown are the mean ± SEM of at least 3 independent experiments.


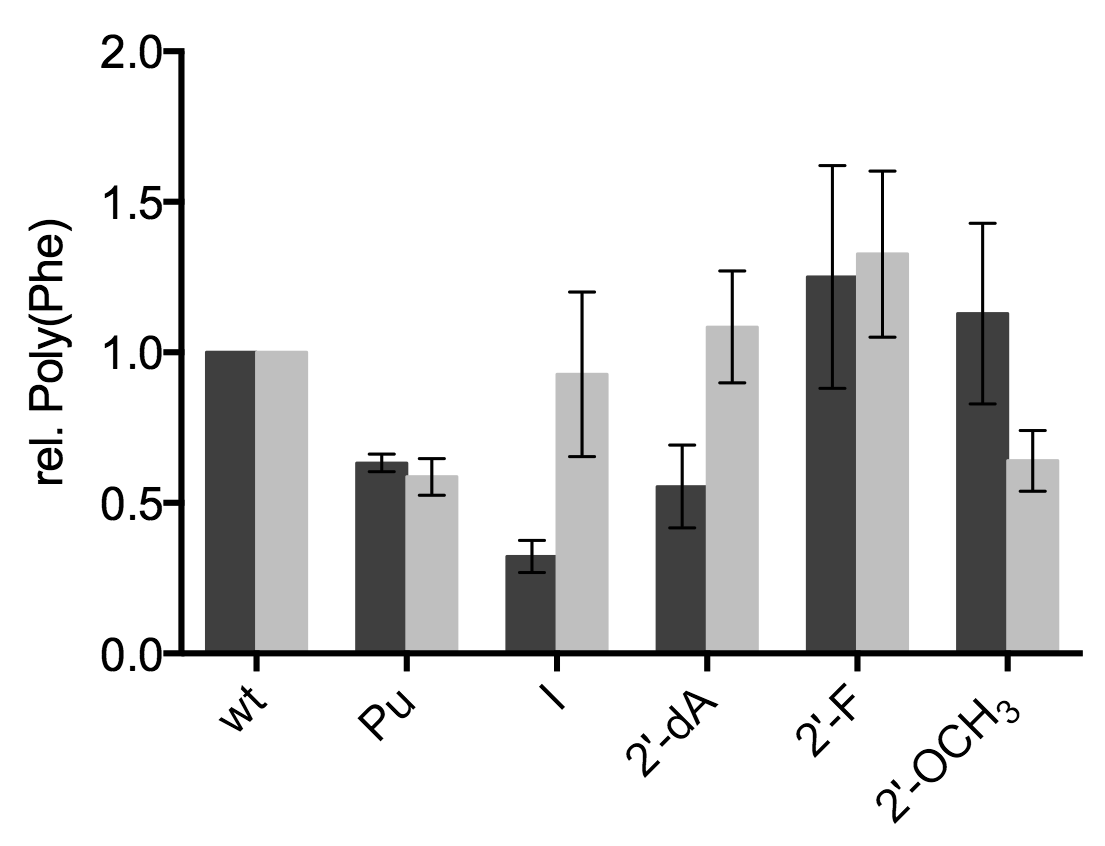


**Supplemental Figure 3**: Translation of a (UUC)_12_ mRNA using ribosomes modified at position 1492 (dark grey) or 1493 (light grey). The activity of ribosomes harboring the unmodifed RNA oligonucleotide was set to 1. The values shown are the mean ± SEM.
